# Effect of obesity and hypertension on left ventricular geometry and function among asymptomatic Indian adults

**DOI:** 10.6026/973206300200862

**Published:** 2024-08-31

**Authors:** Shree C Mamatha, Prashanth KS, Girija B

**Affiliations:** 1 Department of Physiology, Sri Devaraj Urs Medical College, Sri Devaraj Urs Academy of Higher Education and Research, Kolar, Karnataka, India; 2 Department of Physiology, Bangalore Medical College & Research Institute, Bengaluru, Karnataka, India

**Keywords:** Left ventricular diastolic function, left ventricular geometry, left ventricular systolic function, non-obese hypertensive, obese normotensives

## Abstract

Hypertension & obesity are important independent predictors of cardiovascular morbidity & mortality specifically left ventricular (LV) functions. With the Objectives to examine the effect of obesity & hypertension on echocardiographic parameters of geometry, systolic, and diastolic functions of left ventricle in asymptomatic adults the cross-sectional study was conducted with 100 individuals (60 male & 40 female). Their Blood Pressure & Body Mass Index was recorded following which they were divided into obese normotensives & non-obese hypertensive. Echocardiographic parameters indicating LV geometry and function were recorded. Data was statistically analysed with Unpaired t test considering p value <0.05 as significant. LV geometry and LV systolic function parameters were significantly altered in non-obese hypertensive. Parameters of LV diastolic functions were affected in obese normotensives. 54% of obese normotensives had LV diastolic dysfunction & 58% of non-obese hypertensive had LV diastolic dysfunction. 2% of obese non-hypertensive individuals had LV systolic dysfunction & 6% of non-obese hypertensive had LV diastolic dysfunction. Hypertension significantly affects LV geometry, LV systolic and diastolic function. Obesity affects LV geometry & diastolic functions of LV even in individuals with preserved systolic function.

## Background:

Hypertension and obesity are among the most common public health problems in both developed and developing countries. [1] High blood pressure and high body mass index have been shown to be important independent predictors of cardiovascular morbidity and mortality. [2,3] Available literature says that Hypertension over a period of time causes changes in the left ventricular mass and geometry, which result in systolic and diastolic dysfunction. [4] Studies have revealed subclinical diastolic dysfunction in hypertensive in spite of preserved global systolic function. [5] In these patients, hypertension has been implicated in a gradual decline in left ventricular diastolic function, culminating in a state of diastolic heart failure. [5] Obesity tremendously increases metabolic and haemodynamic demand, leading to adaptive alterations in cardiac structure and function. [6] Excessive epicardial fat, adipose tissue, increased free fat mass, increased intravascular volume and greater sympathetic drive are a few of the several factors related to obesity which have been implicated in LV wall stress that predisposes to changes like eccentric left ventricular hypertrophy and diastolic dysfunction. [6,7] Compounding the problem is the fact that changes in LV function have been reported not only in clinically asymptomatic patients, but even in those with normal ejection fraction and preserved global LV systolic function. [8] The above factors emphasize the importance of early detection of changes in LV structure and function in the high risk population, and also the need for a non-invasive tool to achieve this end. [8] Recent techniques have helped in the evaluation of functions of heart in asymptomatic adults. [8] Echocardiography is considered a validated and sensitive technique for measuring and classifying changes in LV structure and functions.[1] Therefore, it is of interest to compare the effects of blood pressure and obesity on Echocardiographically assessed systolic and diastolic functions of left ventricle in asymptomatic adults. We hypothesized that hypertension and/or obesity significantly alter left ventricular functions even in those without clinically evident cardiovascular disease.

## Materials and Methodology:

A cross-sectional study was conducted between May 2018 and July 2018 at the department of Physiology, Bangalore Medical College and Research Institute, and department of cardiology, PMSSY Super-specialty Hospital, attached to the college. Ethical clearance was obtained from institutional ethics committee.

## Sample size was calculated based on previous studies, in consultation with a statistician, using the formula:

n = 2(Zα+ Z1-β)2σ2 / d2 where,

Zα = Alpha Error

Z (1-β) = Beta Error

σ = Standard Deviation

d = Effect Size

One of the parameters used was IVSd where, σ = 1.5, Zα = 1.96, Z (1-β) = 0.84 and d= 0.8 Sample size was calculated as 100 based on previous studies, using appropriate formula. Subjects would then be divided into two groups, based on blood pressure and Body Mass Index (BMI) measurements. The two groups were: obese individuals without hypertension and non-obese individuals with hypertension.

Subjects were chosen by simple random sampling method from the general population of the city, and from among the relatives accompanying the patients of the hospital. Participation was enlisted on voluntary basis. Enrolment into the study was based on the eligibility criteria mentioned below.

## Inclusion criteria:

[1] Individuals of either gender, in the age group of 40 -75 years.

[2] Non-smokers.

[3] Subjects who were previously diagnosed with hypertension, with or without treatment and BMI <25kg/m2 were included in the Hypertensive non-obese group. [9,10]

[4] Subjects who were never before diagnosed with hypertension, and BP <140/90 mm of Hg at the time of examination, with BMI ≥25 kg/m2 were included in Obese normotensive group. [10]

## Exclusion criteria:

[1] Subjects with history suggestive of or diagnosed cardiovascular disorders.

[2] Subjects with history of diabetes mellitus.

[3] Smokers and alcoholics.

[4] Subjects with history of neuromuscular disorders.

[5] Subjects with history of chronic respiratory disorders.

[6] Subjects with history suggestive of or diagnosed endocrinal disorders.

[7] Subjects with complications of prolonged hypertension like nephropathy, neuropathy, peripheral vascular disorders.

[8] Subjects on medication which may affect body weight or have cardio-toxic potential.

[9] Subjects with history of chronic renal disorders.

[10] Subjects with suspected or established secondary hypertension.

[11] Subjects who will be diagnosed with valvular lesions and impaired global/ segmental wall motion during echocardiographic assessment.

Informed written consent was taken from subjects who met the above criteria, and were willing to participate in the study. A detailed general and medical history was taken using pre-structured questionnaire after which they were examined clinically.

## Blood pressure measurement:

Subjects were asked to rest for 5 minutes in supine position & their blood pressure was recorded with sphygmomanometer & stethoscope. Two readings each of systolic and diastolic blood pressure were taken ten minutes apart, by standard procedure, and average of the two readings was considered for tabulation.

## BMI:

Height was measured with wall attached stadiometer. Subject was made to stand barefoot, in erect posture, with heels, gluteus & occiput touching the wall, & height was recorded in centimeters, then converted to meters. Weight was measured using weighing scale in kilograms with subject wearing light clothes without footwear. Using these values BMI was calculated in kg/m2 using the formula BMI = Weight in kgs / square of Height in meters

## Echocardiography:

After recording above mentioned parameters, subjects underwent echocardiography. The procedure was done by experienced technicians in cardiology department of PMSSY super-specialty hospital. GE Vivid E9 ultrasound machine was used. All subjects underwent a standard echocardiographic examination in the left lateral position. The echo/Doppler examination included parasternal long- and short axis views and apical four chamber and five chamber views, and subcostal view or suprasternal view in selected cases. For each view, at least three consecutive cardiac cycles were recorded during quiet respiration. The following parameters were obtained from echocardiography.

## Parameters measuring LV geometry are,

[1] Systolic Inter Ventricular Septal thickness (IVSs in cm)

[2] LV Internal Diameter at end systole (LVIDs in cm)

[3] Diastolic Inter Ventricular Septal thickness (IVSd in cm)

[4] LV Internal Diameter at end diastole (LVIDd in cm)

## Important parameters measured to assess systolic function are,

[1] Ejection systolic volume (ESV in ml)

[2] Systolic Inter Ventricular Septal thickness (IVSs in cm)

[3] Ejection Fraction (EF in %)

[4] LV Internal Diameter at end systole (LVIDs in cm)

## The important parameters to assess diastolic function are,

[1] LV Internal Diameter at end diastole (LVIDd in cm)

[2] Diastolic Inter Ventricular Septal thickness (IVSd in cm)

[3] Ejection Diastolic Volume (EDV in ml)

[4] Deceleration time (DT in ms)

[5] E/A ratio (E- Early Diastolic Filling Velocity, A-Late Diastolic Filling Velocity)

[6] E/é (é - Mitral Annular Velocity)

[7] Iso-Volumetric Relaxation Time (IVRT in ms)

Group values were converted to mean and standard deviation. Unpaired t test was used to compare the different parameters between two study groups, with level of significance at p < 0.05.

## Results:

**Figure 1:**
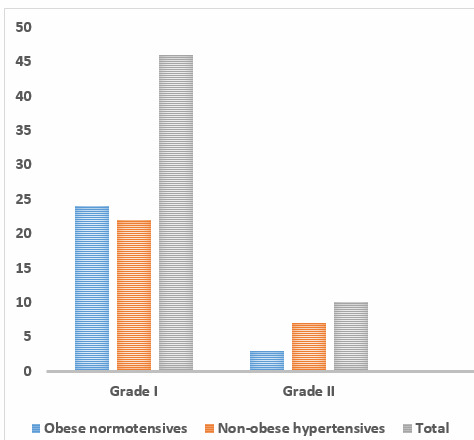
Prevalence of diastolic dysfunction

**Figure 2:**
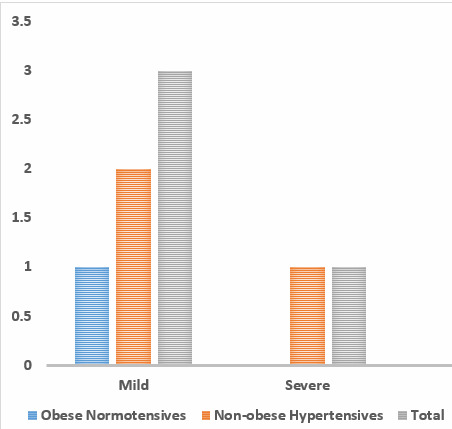
Prevalence of systolic dysfunction

The study was conducted on 100 subjects who were subdivided into two groups. There were 50 subjects in each group. Mean age (in years) of obese normotensives was 52.12 ± 8.6 and non-obese hypertensive was 57.12 ± 11.5. From Figure 1, on graphically representing prevalence of diastolic dysfunction, 54% of obese normotensives and 58% of non-obese hypertensive had LV diastolic dysfunction. From Figure 2, on graphically representing prevalence of systolic dysfunction 2% of obese normotensive individuals and 6% of Non-obese hypertensive had LV systolic dysfunction.

## Discussion:

Obesity and hypertension are the important independent risk factors for cardiovascular morbidity, hence are known to cause subclinical changes in asymptomatic individuals. This study was undertaken to explore their effects on left ventricular structure and function. Echocardiography is a non-invasive, convenient technique and is a reliable tool for assessment of the same. Independent variables in our study were Blood Pressure and BMI. Based on these, the total study population of 100 was divided into two groups of 50 each. One group consisted of hypertensive individuals without obesity & second group of obese individuals without hypertension, as per standard defining values quoted earlier. On comparing obese normotensives with Non-obese Hypertensives, it was established that the former group had greater BMI while the latter group had higher SBP and DBP. Thus, it was established that they had one risk factor each, either hypertension or obesity. Inter-group comparison of ECHO parameters in these subjects made it possible to assess the effect of obesity and hypertensive on left ventricular geometry and function.

From Table 3, in hypertensive group IVSs, IVSd, LVIDs, LVIDd were all significantly higher than in normotensives, indicating that hypertension had a significant effect on LV geometry, predisposing to hypertrophy. ESV was higher and EF lower, hinting towards possible progression to systolic dysfunction. From Table 3, the obese group, despite being normotensive, and having normal EF, had poor EDV, increased DT and IVRT and decreased E/A and E/é, which when interpreted together, are indicative of diastolic dysfunction. The differences in the mean values of each of these compared to the other group, however was not statistically significant. As seen from graph 1, 54% of obese normotensives and 58% of non-obese hypertensives had LV diastolic dysfunction. It was found that the number of subjects falling into categories of mild to moderate diastolic dysfunction was considerable in the study population as a whole, and slightly higher in the Non-obese Hypertensive. Graph 2 shows the prevalence of LV systolic dysfunction among 2 study groups. 2% of obese normotensive individuals and 6% of Non-obese hypertensives had LV systolic dysfunction. Prevalence of LV systolic dysfunction was significantly higher in the Non-obese hypertensives than the other group.

Though many studies have been done, the relationship between LV diastolic dysfunction and hypertension is less clear and remains poorly understood. Few studies have concluded that sub-optimally treated hypertension results in steadily increasing LV end-diastolic pressures and later lead to diastolic heart failure, characterized by limited myocardial relaxation, preserved LVEF, and a significant annual mortality. [11] Geometric alterations progresses to left ventricular dilatation and failure in hypertensives. [12] Adewole *et al.* reported that prevalence of LVH in their study on hypertensives ranged between 30.9-56%. 61%-74% had abnormal LV geometry, commoner in women. Eccentric LV geometry was observed in 17.5-30.4% hypertensives when compared to concentric LV geometry which was reported in 3.3-25.6% of the subjects. Normal geometry was seen in 26-38.9%. Wachtell *et al.* reported prevalence of LVH in 42-78% hypertensives and 63-86% had abnormal LV geometry. [9]

Waala *et al.* reported that obesity is associated with increased aortic diameter, left atrial enlargement, and increased LV mass, with increased septal wall and posterior wall thickness. Zarich *et al.* observed that the values for active mitral filling (A) were not significantly affected, but there was a significant decrease in the maximum velocity of passive mitral filling (E) among obese patients, resulting in a decrease in the E/A ratio. Conversely, Chakko *et al.* found that the values of A were increased, but no significant differences in the values of E were noted, resulting in a decreased E/A ratio. Stoddard *et al.*, in their study on obese subjects, found a significant increase in both E and A values, so the E/A ratio were unaltered [13]. Bindu Garg found that, 53% overweight subjects and 35% obese subjects had diastolic dysfunction with normal ejection fraction. [10] Evrim *et al.* & several other studies have found that LV ejection fraction is normal to increase in majority of the obese subjects. [14]

## Implications:

The study goes to prove that obesity and hypertension can deleteriously affect LV structure and function. These effects may be present in asymptomatic individuals in the form of progressive worsening of diastolic function, which may again remain sub-clinical and undetected for a long time. Echocardiography is a non-invasive and reliable tool for assessment of LV geometry and function and may be productively employed in the population at risk of cardiovascular morbidity, for early detection of changes before they produce symptoms. As both hypertension and obesity are modifiable risk factors, suitable and early steps taken to control these will go a long way in preserving and promoting cardiovascular health.

## Conclusion:

Hypertension significantly affects LV geometry, LV systolic and diastolic function. Obesity affects LV geometry & diastolic functions of LV even in individuals with preserved systolic function.

## Source of Funding:

Self

## Figures and Tables

**Table 1:** Comparison of weight & BMI between obese normotensives & non-obese hypertensive

	**obese normotensives**	**non-obese hypertensive**	**P value**
Weight (kg)	73.2 ± 14.07	56 ± 9.5	<0.001
BMI (kg/m^2^)	29.56 ± 3.47	21.58 ± 2.9	<0.001

**Table 2:** Comparison of Blood Pressure between obese normotensives & non-obese hypertensive

	**obese normotensives**	**non-obese hypertensive**	**P value**
SBP(mmHg)	135 ± 13.1	141.96 ±17.8	<0.001
DBP(mmHg)	83.2 ± 7.94	90.64 ± 10.1	<0.001

**Table 3:** Comparison of echo parameters between obese normotensives & non-obese hypertensive

	**Obese normotensives**	**non-obese Hypertensive**	**P value**
ESV (ml)	34.8 ± 8.48	41.31 ± 18.36	0.01
IVSs (cm)	1.19 ± 0.05	1.27 ± 0.16	<0.001
EF (%)	62.7 ± 11.04	58.7 ± 6.41	0.01
LVIDs (cm)	2.94 ± 0.27	3.13 ± 0.51	0.01
LVIDd (cm)	4.39 ± 0.36	4.57 ± 0.5	0.02
IVSd (cm)	0.9 ± 0.07	0.98 ± 0.15	0.001
EDV (m)	90.26 ± 18.27	99.3 ± 26.67	0.02
DT (ms)	231.98 ± 43.08	225.6 ± 47.3	0.24
E/A ratio	0.96 ± 0.32	1.01 ± 0.41	0.24
E/eé	6.78 ± 1.94	7.85 ± 2.89	0.01
IVRT (ms)	95.4 ± 11.7	93.4 ± 13.4	0.2
